# Which is the better timing between embolization and surgery for hypervascular spinal tumors, the same day or the next day? A retrospective comparative study

**DOI:** 10.1097/MD.0000000000010912

**Published:** 2018-06-18

**Authors:** Benqiang Tang, Tao Ji, Wei Guo, Xiaodong Tang, Long Jin, Sen Dong, Lu Xie

**Affiliations:** a Musculoskeletal Tumor Center, People's Hospital, Peking University; b Department of Spine Surgery, Beijing Luhe Hospital, Capital Medical University; c Department of Radiology, People's Hospital, Peking University, Beijing, China.

**Keywords:** hypervascular tumor, intraoperative blood loss, preoperative embolization, spinal tumor, spine surgery

## Abstract

Previous series presented the timing between embolization and surgery in a wide range on the basis of their experience rather than supportive data. And comparative studies were limited to small samples. In addition, there is no study publishing the timing by considering both safety and efficacy of embolization. The aim of this study was to determine the better timing (the same day or the next day) between embolization and surgery for hypervascular spinal tumors by assessing the safety and efficacy of embolization.

One hundred twenty-five embolizations with subsequent 120 operations for hypervascular spinal tumors between January 2010 and April 2013 were retrospectively reviewed. The time between embolization and surgery was mainly determined by interventional radiologist schedules and operating room available. Major complications of embolization were documented. The efficacy of embolization was compared between the same day and the next day group.

Of the 125 embolizations, there were 4 major complications, all of which occurred on the same day of procedure. Of the 120 operations, 36 cases were operated on the same day of embolization, 74 on the next day, and 10 on the second day. When comparing the efficacy of embolization between the same day and the next day group, intraoperative blood loss (1483 ± 1475 vs 1548 ± 1099 mL, *P* = .80), intraoperative transfusion requirement (1011 ± 1200 vs 1112 ± 890 mL, *P* = .62), and postoperative blood loss (1146 ± 933 vs 1031 ± 777 mL, *P* = .50) were not significantly different.

Embolization carries certain risks (4/125, 3.2%) for major complications, which may occur within the time window of 1 day. Two patient groups showed no difference on the efficacy of embolization. Operation should be scheduled on the next day of embolization if possible.

## 1 Introduction

Angiographic hypervascularity was reported in many metastatic and primary tumors in spine.^[1–15]^ As an adjuvant technique, preoperative embolization has been demonstrated the favorable outcome in reducing intraoperative blood loss for hypervascular lesions.^[6,9,11,16–18]^ Some known factors that impact the efficacy of embolization include histology, extent of tumor or tumor volume, the completeness of embolization, and the complexity or the invasiveness of the surgery.^[19–21]^ The time between embolization and surgery has also been discussed as one potential factor on the efficacy of procedure.^[3]^

Many authors suggested that surgery should be performed within 24 hours on the basis of their experience rather than supportive data.^[10,16,22]^ In addition, there have been conflicting reports on the hypothesis that the sooner the surgery is performed, the better the outcome would be.^[3,23,24]^ What is more, previous studies presented the timing by only considering the efficacy of embolization; however, to our knowledge, the safety of embolization should be another main concern.

The purpose of the comparative study, with largest cases to date, was to determine the better timing (the same day or the next day) between embolization and surgery for hypervascular spinal tumors by assessing the safety of embolization in terms of its major complications, and by comparing the efficacy of embolization in 2 patient groups on the basis of different time span.

## 2 Materials and methods

Approval for this retrospective study was obtained from the Institutional Review Board of People's Hospital Peking University. The need for informed consent was waived because of the retrospective study design. All the data were collected and analyzed anonymously.

### 2.1 Data review

This is a comparative study by retrospectively collected data. A total of 130 consecutive angiographies for spinal tumors were identified between January 2010 and April 2013 at our database. The time between embolization and surgery was mainly determined by interventional radiologist schedules and operating room available. Angiography and embolization were mainly indicated for renal cell cancer and thyroid gland cancer, metastatic lesions that are highly suspicious of hypervascularity from previous personal or reported experience, or available imaging findings, primary tumors with known hypervascularity, such as giant cell tumor and aneurysmal bone cyst, and spinal neoplasms for which complex or extensive surgery is planned. And the decision for embolization was made as the operating surgeon's personal experience and preference. Approximately, embolization was performed in only 10% of all spinal tumor patients who received surgical treatment at our institution. Of note, the indication of embolization varied among centers.^[1–3,5–7,10,18,19]^ The degree of vascularity of the tumor was categorized subjectively by 2 senior interventional radiologists as hypovascular (tumor blush less than normal vertebral body, or avascularity), normal (tumor blush equal to normal vertebral body), and hypervascular (with enlarged and/or increased number of feeding vessels). Once the tumor was identified as hypervascular by angiography, complete (≥90% decrease in tumor blush) or partial embolization (<90% decrease in tumor blush) was performed by 1 of 2 senior radiologists. Embolic agents were gelatin sponge, coils, or a combination. All angiograms and procedure records were reviewed. We excluded the case if the tumor was lack of hypervascularity indicated by angiography.

A total of 120 operations subsequent to 125 embolizations were performed by 4 senior surgeons. All surgical records were reviewed. The following variables that may affect the intraoperative blood loss were registered: age at operation, gender, tumor histology, use of adjuvant radiotherapy or chemotherapy (yes/no), tumor progression or anatomical classification (intracompartmental, extracompartmental, or multiple vertebrae), soft tissue involvement (yes/no), neurological involvement (yes/no), initial operation or reoperation, the number of arteries embolized, embolic agents (coils, gelatin particles, or combined), complete or partial embolization, surgical site (cervical, thoracic, or lumbar), surgical tactics (wide excision, intralesional excision, or palliative decompression), surgical approach (anterior, posterior, or combined), operative time, and the score of surgical invasiveness index (SII).

Of note, the SII is a recently developed method of describing the extent of surgical intervention.^[25]^ It is defined as the sum, across all vertebral levels, of 6 weighted surgical components: anterior decompression (ad), anterior fusion (af), anterior instrumentation (ai), posterior decompression (pd), posterior fusion (pf), and posterior instrumentation (pi). The weights for each component represent the number of vertebral levels at which each of it is performed. A higher score indicates greater invasiveness. For example, for an L1 metastasis with L1 dorsal decompression and pedicle screws bilaterally at T11, T12, L2, and L3, the score is 10 [pd = 1 (1 level) + pf = 5 (5 levels fused) + pi = 4 (instrumentation at 4 levels)].

### 2.2 Procedural safety measures

In this study, the safety of embolization was evaluated on the basis of procedural major complications. Major complications were defined as those adverse events that led to permanent detrimental effect or delay (or cancellation) of the planned surgery (e.g., cerebral infarction, cord ischemia, and vessel dissection or rupture). Adverse events with transient detrimental effect or limited need for further intervention were deemed minor complications (e.g., pain at the site of embolization and low-grade fever).

### 2.3 Procedural efficacy measures

The efficacy of embolization between the same day and the next day group was compared. It was assessed on the basis of data regarding intraoperative blood loss, intraoperative transfusion requirement, and postoperative blood loss. Intraoperative blood loss was calculated as the sum of the soaked in sponge and the collected in suction, intraoperative transfusion requirement as the sum of packed red cell and plasma transfused during operation, and postoperative blood loss as the total drainage from the wound closure to the drainage tube removed. Of note, the indication of intraoperative transfusion was tailored to multiple factors (e.g., hemoglobin level, anesthetists’ preference, and surgeons’ experience).

### 2.4 Statistical analysis

The Chi-square test or Fisher exact test was used to analyze the difference in categorical data between the same-day and the next-day group, and 2-example Student *t* test or Mann–Whitney test in continuous data. The level of significance was a probability value of < .05. The Statistical Package for Social Sciences (SPSS, Chicago, IL; version 17.0) was used for the analysis of the data.

## 3 Results

### 3.1 Safety of embolization

Of the 130 consecutive angiographies, 5 cases were excluded because there was lack of hypervascularity on the angiograms. The remaining 125 cases met the inclusive criteria to analyze the safety of the embolization. Complete embolization was achieved in 116 cases (93%), and near-complete embolization in 9 cases (7%). A combination of sponge and coils was used in 119 cases (95%), and sponge or coils alone in 6 patients (5%). Patient demographic data is presented in Table 1. And histology of the 2 groups is summarized in Table 2.

**Table 1:**
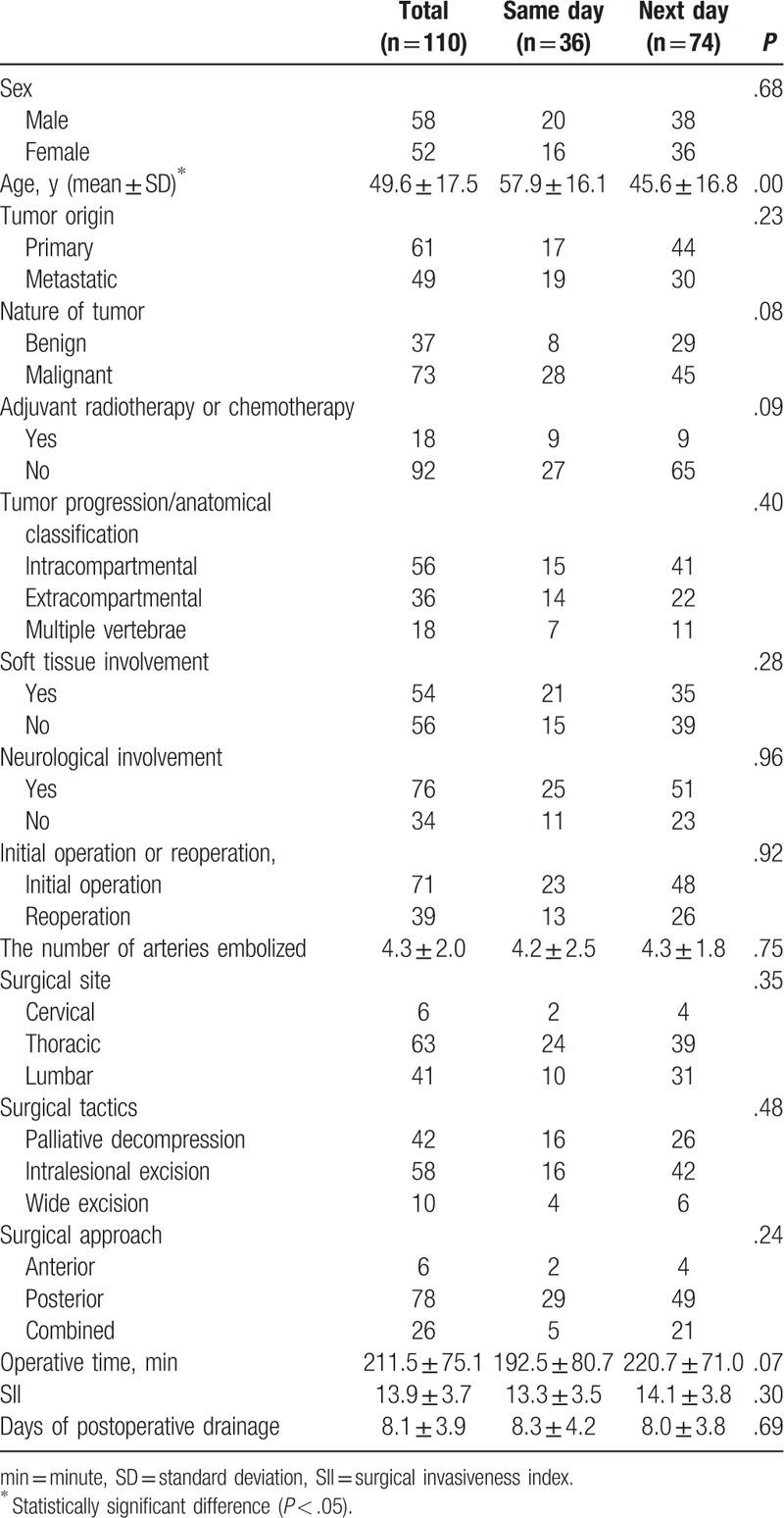
Comparison of variables between the same-day group and the next-day group.

**Table 2:**
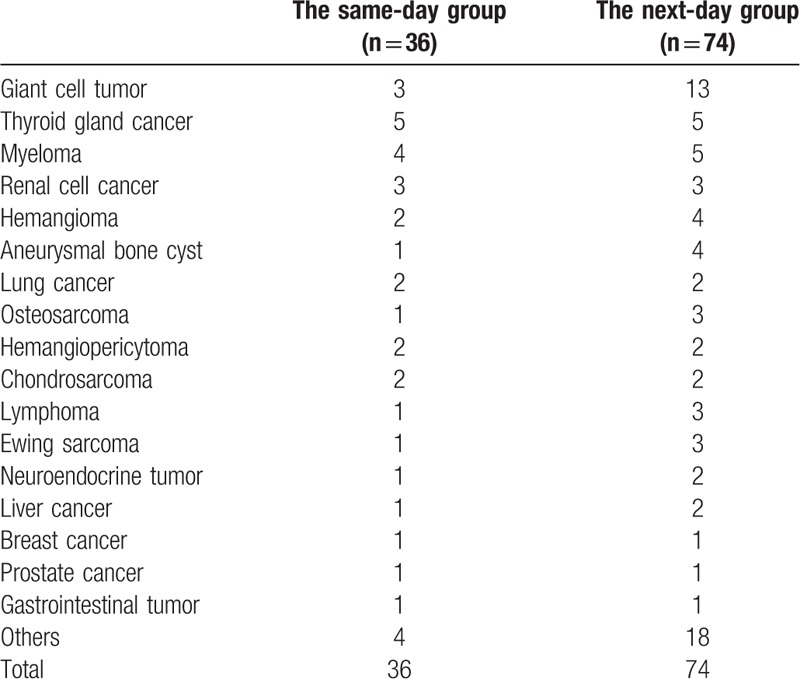
Distribution of tumor histology in 2 groups.

Of the 125 embolizations, there were 4 major complications (3.2% prevalence) in 4 patients. And all the 4 complications were detected on the same day of embolization. One severe hypertension occurred during embolization in a patient for whom planned operation was cancelled. One transient visual field deficit occurred 2 hours after embolization in a patient whose operation was delayed 1 month later. One cerebral embolism occurred 8 hours after embolization in a patient whose operation was delayed 1 month later as well. One cord ischemia was postoperatively identified 2 hours after operation (6 hours after embolization) by retrospective analysis of the angiogram. In this patient, the neurologic deficit may had been ignored or had not appeared before operation due to a narrow time interval between embolization and operation, as operation was performed immediately after embolization. Of note, all the 4 patients had no comorbidities previously. Those were presented in Table 3.

**Table 3:**
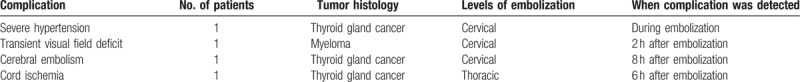
Complications of the 125 embolization procedures.

### 3.2 Efficacy of embolization

Of the 125 embolizations, 2 cases were embolized twice before operation, and 3 cases were excluded because operations were cancelled or delayed. Totally, 120 operations met the inclusive criteria in the analysis of efficacy of embolization. Of the 120 operations, 36 cases were operated on the same day of emboliation, 74 on the next day, and 10 on the second day. The efficacy of embolization between the same day and the next day group was compared.

Of note, tumors were categorized as primary or metastatic and benign or malignant, as the wide spectrum of histology was limited to valid analysis. In addition, degree of embolization and embolic agents were not compared between 2 groups, as the number of subsets was too little to analyze. All baseline data, including patient characteristics, tumor characteristics, embolization-related variables, and surgery-related variables, are summarized between 2 groups in Table 1. All variables, except age, were not significantly different between the 2 groups.

The comparison of the efficacy of embolization was summarized. The intraoperative blood loss was not significantly different between the same-day and the next-day group (1483 ± 1475 vs 1548 ± 1099 mL, *P* = .80). And there was no significant difference in intraoperative transfusion requirement between the 2 groups (1011 ± 1200 vs 1112 ± 890 mL, *P* = .62), nor was postoperative blood loss (1146 ± 933 vs 1031 ± 777 mL, *P* = .50). Of note, mean intraoperative blood loss, intraoperative transfusion requirement, and postoperative blood loss were quite similar between the 2 groups, respectively (Table 4).

**Table 4:**
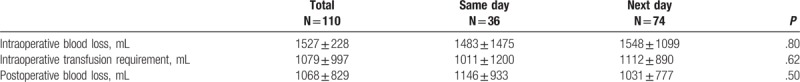
Comparison of the efficacy of embolization between the same-day group and the next-day group.

## 4 Discussion

Although many studies proved that embolization is a safe procedure, ^[1,3,5,17,18,20]^ it does carry certain risks for major complication. In the current study, the rate of major complications, 3.2% (4/125), was comparable to those published ones varying from 1% to 8.5%.^[2,4,8,9,21,24,26]^ Procedural complications may be detected during the embolization, or minutes or hours after embolization. Finstein et al^[15]^ reported that 1 patient complained of gastrocnemius/soleus spasms during the procedure and soon developed complete paralysis due to spinal infarction after embolization. Similarly, Cloft et al^[27]^ presented 1 patient complained of pain in lower back and both legs during embolization and progressively developed paralysis over 12 hours after embolization. However, Fernandez-Torron et al^[28]^ demonstrated that a patient had no complaint during the embolization but developed a Brown–sequard syndrome 15 minutes after embolization. And Kobayashi et al^[21]^ reported 2 patients developed neurologic deficits 2 and 6 hours after embolization, respectively. Similarly, Smith et al^[29]^ presented 1 patient who experienced progression of lower limb weakness several hours after embolization. In this study, 1 severe hypertension, 1 transient visual field deficit, 1 cerebral embolism, and 1 cord ischemia occurred during embolization, 2, 8, 6 hours after embolization, respectively. When surgery is scheduled on the same day, it is less likely to detect those major complications because of a narrower time span. And, if a complication was not noticed before surgery, it then would be deemed to be one arisen from surgery. Surgeons should wait for surgery on the following day to assess and treat any major complications of embolization. Of note, in our experience, embolization in cervical spine tumors carries a higher risk for complication than in thoracic or lumbar spine, due to frequent anastomoses between carotid, vertebral, and subclavian arteries.

Many authors recommended the optimal time would be immediately after embolization, as the main concern is recanalization and collateral establishment.^[3,10,30]^ However, in many comparative studies, the effect of a time lapse (e.g., immediate vs delayed, ≤ 24 vs > 24 hours, < 48 vs ≥ 48 hours) on the intraoperative blood loss seems to be not significant. Of note, most of the cases in those studies were operated within 72 hours after embolization. One study showed a similar effect on intraoperative blood loss between immediate group and delayed group.^[23]^ Two studies presented that the same-day group and the next-day group, or ≤ 24 hours group and > 24 hours group, had no significant difference in the intraoperative blood loss.^[3,18]^ Similarly, in another 2 studies, there was no significant difference in intraoperative blood loss between < 48 hours group and ≥ 48 hours group.^[8,24]^ Those are presented in Table 5. According to those published results, we speculated that effectiveness of embolization may not become weaker over time when surgery plan was restricted within 48 to 72 hours after embolization. Indeed, many studies showed that intraoperative blood loss tend to be larger when surgery was performed more than 72 hours after embolization.^[31,32]^ The reason would be that recanalization and collateral establishment happen in a later stage, which may be within 2 to 3 days, rather than within hours. Similarly, we did not find the reduction of effectiveness due to the delay of surgery in the next day in comparison to the same day group. However, we think embolization would gradually lost the effectiveness over days. In order to gain the maximal devascularization effect of embolization and maximal probability to detect potential major complications, we advocate embolization should be scheduled on the next day of embolization.

**Table 5:**
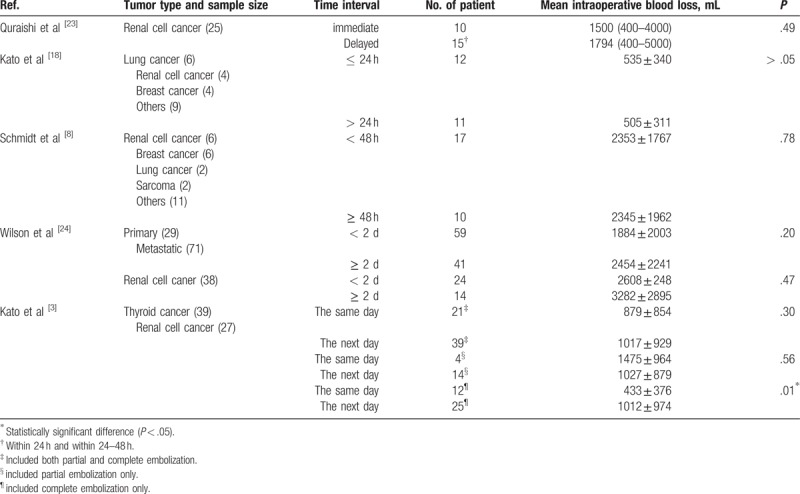
Summary of comparative studies on the efficacy of embolization based on the different time interval.

Of note, Kato et al^[3]^ concluded that surgery should be scheduled on the same day of embolization, as a subgroup analysis of complete embolization (n = 37) revealed that intraoperative blood loss was significantly larger in the next day group in comparison to the same-day group. However, the small sample of 37 cases limited the strength of their study. And, many authors showed that degree of the embolization (complete vs partial) has a limited effect on the intraoperative bold loss.^[5,18,21,24]^

One biggest advantage of the study is the methodology that not only efficacy of embolization but also safety of embolization were considered when determining the better time between embolization and surgery. Surgical series only included patients who underwent embolization and subsequent surgery, as a patient who may have had a complication after embolization and then did not undergo surgery would be excluded from analysis in those studies. However, we think the potential complications should be one of the main concerns on the timing. The second advantage of the study is that all the parameters, except age, did not significantly differ between the 2 groups. Of note, the mean of arteries embolized was nearly the same between the 2 groups (4.2 ± 2.5 vs 4.3 ± 1.8). In addition, the SII (13.3 ± 3.5 vs 14.1 ± 3.8), as well as operative time (192.5 ± 80.7 vs 220.7 ± 71.0 m), was quite similar between the 2 groups. Those indicated the strength of this comparative study. In addition, to our knowledge, this is a largest comparative study to date.

The limitations to our study are primarily a result of its retrospective design. The time between embolization and surgery was not randomly assigned, and hence, many confounding variables may modify the results on the comparison of the efficacy between 2 groups. A randomized controlled study is needed to drive firm conclusions, despite of the time constraint of resources with the angiography suite, anesthesia, the operating room, and surgeon availability. Another limitation of the study was that a wide range of tumor pathologies was enrolled. A cohort of the homogeneous histology can reach clear finding; however, it is difficult to collect such a sample big enough to reach analysis. What is more, the intraoperative transfusion was not indicated by the same criterion, which makes it a weaker estimated value. Hence, in the current study, the better timing between embolization and surgery has to be interpreted with caution.

Despite these limitations, our findings have interesting clinical implications and should be kept in consideration.

## 5 Conclusion

The current study indicated that major complications of embolization may occur within the time window of 1 day. Despite the incidence of major complications was only 3.2%, the surgery should be scheduled for the next day to allow the major complications of embolization to be detected and treated so that they would not be misdiagnosed as the surgical complications. In addition, in the current study, we did not find the reduction of effectiveness due to the delay of surgery on the next day in comparison to the same-day group. In summary, operation should be scheduled on the next day of embolization if possible.

## Author contributions

**Conceptualization:** Benqiang Tang.

**Data curation:** Tao Ji, Xiaodong Tang, long jin.

**Formal analysis:** Benqiang Tang, Sen Dong.

**Methodology:** Benqiang Tang, Xiaodong Tang.

**Software:** Tao Ji.

**Supervision:** Wei Guo.

**Writing – original draft:** Benqiang Tang, Lu Xie.

**Writing – review & editing:** Benqiang Tang, Tao Ji.
